# Predictive Value of Left Atrial and Ventricular Strain for the Detection of Atrial Fibrillation in Patients With Cryptogenic Stroke

**DOI:** 10.3389/fcvm.2022.869076

**Published:** 2022-04-25

**Authors:** Gabriella Bufano, Francesco Radico, Carolina D'Angelo, Francesca Pierfelice, Maria Vittoria De Angelis, Massimiliano Faustino, Sante Donato Pierdomenico, Sabina Gallina, Giulia Renda

**Affiliations:** ^1^Department of Innovative Technologies in Medicine & Dentistry, Institute of Cardiology, G. d'Annunzio University Chieti-Pescara, Chieti, Italy; ^2^Department of Cardiology, Renzetti Hospital, Lanciano, Italy; ^3^Department of Neurology, Stroke Unit, SS Annunziata Hospital, Chieti, Italy; ^4^Department of Cardiology, SS Annunziata Hospital, Chieti, Italy; ^5^Department of Neuroscience, Imaging and Clinical Sciences, Institute of Cardiology, G. d'Annunzio University Chieti-Pescara, Chieti, Italy

**Keywords:** atrial fibrillation, cryptogenic stroke, insertable cardiac monitor, atrial myopathy, left atrial strain, left ventricular longitudinal strain

## Abstract

**Background and Aims:**

Cryptogenic stroke (CS) is associated with a high rate of recurrences and adverse outcomes at long-term follow-up, especially due to its unknown etiology that often leads to ineffective secondary prevention. Asymptomatic atrial fibrillation (AF) could play an important pathophysiological role. Some studies have pointed to left atrial (LA) and left ventricular (LV) systolic and diastolic dysfunction as surrogate markers of AF. The aim of the study is to evaluate the relationship between echocardiographic parameters of LA and LV function, and the occurrence of AF revealed by continuous ECG monitoring in a cohort of patients with CS.

**Methods:**

Single-center prospective cohort study. Seventy-two patients with CS with insertable cardiac monitors (ICM) underwent transthoracic echocardiography (TTE). TTE was focused on LA and LV function, including both standard and longitudinal strain-derived parameters. All detected AF episodes lasting more than 2 min were considered.

**Results:**

Continuous ECG monitoring revealed subclinical AF in 23 patients (32%) at an average of 6.5 months after ICM implantation. Many echocardiographic parameters, indicating LA volume and LV systolic/diastolic function, were significantly associated with the occurrence of AF, suggesting the worst atrial function in the AF group. Furthermore, multivariable regression analysis revealed that peak atrial contraction strain and left ventricular strain were independently associated with AF (adjusted OR = 0.72, CI 95% 0.48–0.90, *p* = 0.005, and adjusted OR = 0.69, CI 95% 0.46–0.95, *p* = 0.041, respectively).

**Conclusion:**

In patients with CS, LA and LV strain analysis add predictive value for the occurrence of AF over clinical and morpho-functional echocardiographic parameters. Impaired booster pump strain and LV longitudinal strain are strong and independent predictors of AF.

## Introduction

Cryptogenic stroke (CS) is defined as cerebral ischemia of undetermined origin (1). About 87% of strokes are of ischaemic origin, and 20–30% of ischaemic strokes are estimated to be cryptogenic or of undetermined cause (2). The dominant underlying mechanism of CS seems to be an embolism from an unestablished source. Therefore, the term Embolic Stroke of Undetermined Source (ESUS) was introduced to define such a subset of CS (3). ESUS may account for different embolic sources: atrial cardiopathy, hidden atrial fibrillation (AF), left ventricular (LV) disease, atherosclerotic plaques, patent foramen ovale (PFO), cardiac valvular disease, and cancer (4). Several trials revealed that hidden AF could be detected in up to 30% of patients with ESUS at long term follow-up (5–7). However, its causal association with the index stroke remains a matter of debate (4). Furthermore, two randomized controlled trials (8, 9) failed to demonstrate the superiority of oral anticoagulation over aspirin for the prevention of stroke recurrence in patients with ESUS, probably because of the wide heterogeneity of the patients included in the ESUS definition. Accordingly, early diagnosis of stroke etiology is crucial in order to evaluate the effective strategies in secondary prevention and to particularly identify patients who may best benefit from oral anticoagulation, such as those with hidden AF. For this reason, the use of an Insertable Cardiac Monitor (ICM) has been proposed to enhance the detection of AF and improve the prognosis in patients with CS (10). Specific CS subgroups like older patients (8) or patients with high CHA_2_DS_2_-VASC score (10) portend a higher probability of hidden AF and could take advantage of long-term ECG monitoring. Since the substrate for AF relates to left atrial (LA) dilation and fibrosis, with subsequent structural and electrophysiological remodeling, dysfunction, and delay in electromechanical conduction (11), echocardiographic parameters investigating LA size and function are gaining growing interest as a surrogate predictor of hidden AF (12, 13), even more so in patients with CS (14, 15). Moreover, the assessment of LV diastolic parameters and compliance, also affecting LA size and function (16–18), can be of relevance in this setting (19, 20).

Some studies have pointed LA dysfunction as a surrogate predictor of hidden AF in patients with CS, but the assessment of incident AF was heterogeneous, mostly based on clinical follow-up, and were more frequently short-term, with a small percentage of patients implanted with cardiac loop recorder (21, 22). This approach could underestimate the incidence of AF after CS, therefore underestimating the relationship between LA dysfunction and AF.

The aim of this study was to evaluate the relationship between echocardiographic parameters, indicating LA and LV function, and the occurrence of AF revealed by continuous ECG monitoring after CS. Particularly, we investigated the predictive value of LA strain and function and LV diastolic function for the detection of AF episodes.

## Materials and Methods

This is a single-center prospective cohort study. We enrolled consecutive patients from March 2016 to September 2020 who were admitted to the Neurological Clinic of Santissima Annunziata Hospital of Chieti with the diagnosis of CS. The etiology was determined by the neurologist according to Trial of Org 10172 in Acute Stroke Treatment's (TOAST's) classification (1), which states that a stroke was classified as “cryptogenic” if extensive testing failed to reveal a clear etiology. The workup included: 24-h ECG monitoring, standard transthoracic echocardiography, screening for thrombophilic states (in patients under 55 years of age), transcranial and neck Doppler ultrasound, computed tomography (CT) angiography of the head and neck, and magnetic resonance angiography in patients with negative CT findings. We collected clinical parameters such as physical characteristics, medical history, and cardiovascular risks factors. A long-term electrocardiographic monitoring insertable system was planned in all patients with CS, and an echocardiographic assessment, including both standard and strain-derived parameters, was performed. Follow-up clinical events (all-cause death, cardiovascular death, stroke, myocardial infarction, bleeding) were reported by local investigators after verification from clinical records and source documents, or in case of missing data by a phone questionnaire.

### Echocardiographic Assessment

Echocardiographic examinations were performed within 30 days from the index stroke using a commercially available ultrasonography system (Philips Affinity 50c). Standard echocardiographic parameters were recorded and measured while blinded to cardiac monitoring findings (G. B.) and according to the recommendations of international guidelines (23), including LA volume, LV mass, and LV systolic/diastolic function. All volumetric measures were indexed to body surface area (BSA). Other echocardiographic parameters included in analysis were as follows: maximum (max), minimum (min), and pre-atrial (Pre-A) LA contraction volume. These parameters were recorded from the apical 4-chamber (A4-C) and 2-chamber (A2-C) views as follows: maximum LA volume (LAV max), measured on the 2D frame just before mitral valve opening, LA pre-atrial contraction volume (LAV pre A), measured on the frame just before the onset of atrial emptying, and LA minimum volume (LAV min) measured on the frame at end-diastole with the smallest LA volume. All measures were computed separately following American Society of Echocardiography guidelines, and using the biplane modified Simpson's method of discs.

Both apical views were optimized in terms of orientation, depth, and gain to avoid LA foreshortening and to visualize the entire LA throughout the cardiac cycle. Five cardiac cycles of each plane were stored in cine loop format in order to subsequently select the images of better quality for off-line speckle-tracking analysis.

With these parameters we then calculated volumetric LA function as follows:

Passive LA emptying fraction (LAPEF) (in %): 100 · [LAV max × LAV Pre-A]/LAV max;

Active LA emptying fraction (LAAEF) (in %): 100 · [LAV Pre-A × LAV min]/LAV Pre-A;

Total LA emptying fraction (LATEF) (in %): 100 · [LAV max × LAV min]/LAV max.

Atrial strain was analyzed using A4-C and A2-C views with a frame rate between 70 and 100 frames/s, following European Association of Cardiovascular Imaging (EACVI)/American Society of Echocardiography (ASE)'s consensus document (24). Strain data were digitally stored for offline analysis with Siemens syngo^®^ Vector Velocity Imaging (VVI) Longitudinal version 2.0, with the onset of QRS complex used as the zero-reference point (R-R gating). LA endocardial border was manually traced in both A4-C and A2-C views, delineating a region of interest (ROI) composed by six segments for each view. Then, after the segmental tracking quality analysis and the eventual manual adjustment of the ROI, the longitudinal strain curves were generated by the software for each atrial segment. The resulting atrial strain curve provided 2 peaks consistent with reservoir and contractile strain (24). LA contractile strain was manually calculated from the longitudinal strain mean curve. LA strain pattern consists of a positive wave that peaks at the end of ventricular systole, followed by a decrease after the opening of the mitral valve and, after a plateau, by a second positive wave that corresponds to atrial contraction. From the average of the strain curves, we calculated peak LA strain of A4-C and A2-C view at the end of ventricular systole (peak atrial longitudinal strain, PALS), which is a measure of LA reservoir function (13), and peak atrial contraction strain (PACS), which can be considered a marker of LA pump function, as previously described (13, 16). Passive emptying (conduit) strain was calculated as the difference between PALS and PACS. LV speckle tracking was examined from A4-C, A2-C, and three-chamber (A3-C) view according to EACVI/ASE's position paper (23). In these projections, the software automatically divides each ventricular wall into three segments. From each segment, curves for longitudinal strain were generated (25). In the presence of inadequate view or suboptimal image quality, or when at least one segment of A2-C or A3-C was not correctly visualized, we used only the A4-C view (26).

Patients without interpretable images were excluded from the study. If more than two segments were excluded in a projection, the investigation was deemed unsatisfactory. In all patients, conventional and strain parameters were obtained in sinus rhythm at baseline and without significant valvular heart disease at time of the exam.

Reproducibility of strain measures were confirmed by two physicians (G. B., F. R.) in order to minimize interpersonal variability. The reproducibility was tested while blinded in a random sample of 15 patients, according to a recent practical guideline (27).

### Insertable Cardiac Monitor

Long term cardiac monitoring was obtained in all the study patients for up to 3 years by the Reveal LINQ™ (Medtronic, Dublin, Ireland) ICM. The device was implanted subcutaneously on the thoracic surface. We considered for the analysis all detected AF episodes, defined as an irregular supraventricular rhythm with absence of P waves and variable R-R interval lasting more than 2 min. All recorded AF episodes were evaluated and confirmed by an expert cardiologist (M. F.). AF burden was considered as the number of AF episodes recorded by ICM lasting more than 2 min.

### Statistical Analysis

Categorical variables are described as absolute frequencies and percentages. Continuous variables are presented as mean values ± standard deviation (SD), their associated 95% confidence intervals (CI), or as median and interquartile range (IQR), as appropriate. Normality of variable distributions was tested by the Kolmogorov–Smirnov test. The unpaired Student's *t*-test and chi-squared test were used to compare means and frequencies of clinical and echocardiographic covariates between groups of subjects with or without AF revealed at ICM. Covariates were screened in univariate models by logistic regression to test AF prediction. Then, a multivariable logistic regression analysis was performed to identify independent predictors by selecting covariates with a *p*-value <0.10 at univariate analysis. Kaplan-Meier analysis was performed using Log-rank test in order to compare AF probabilities between patients with or without impaired LVLS and PACS, with discriminative cut-off values for each variable deriving from ROC curve analysis. A ROC analysis was also performed by combining LVLS and PACS. At this scope, a binary logistic regression has been run to generate a probability variable of AF with LVLS and PACS as covariates. Then, a ROC curve was generated by using the probability as the test variable and AF as dependent variable.

Inter and intra-operator reproducibility was assessed by Spearman's correlation coefficient (*r*) and intraclass correlation coefficient (ICC), while agreement was visually assessed by Bland–Altman Plot for inter-operator reproducibility (27).

All probability values were reported as two-sided, and a *p*-value <0.05 was considered significant. Data were processed using IBM SPSS Statistics, Version 25.0. Armonk, NY: IBM Corp.

The study was conducted according to the guidelines of the Declaration of Helsinki and approved by the Institutional Ethics Committee of G. d'Annunzio University/Santissima Annunziata Hospital of Chieti.

## Results

**Figure 1:**
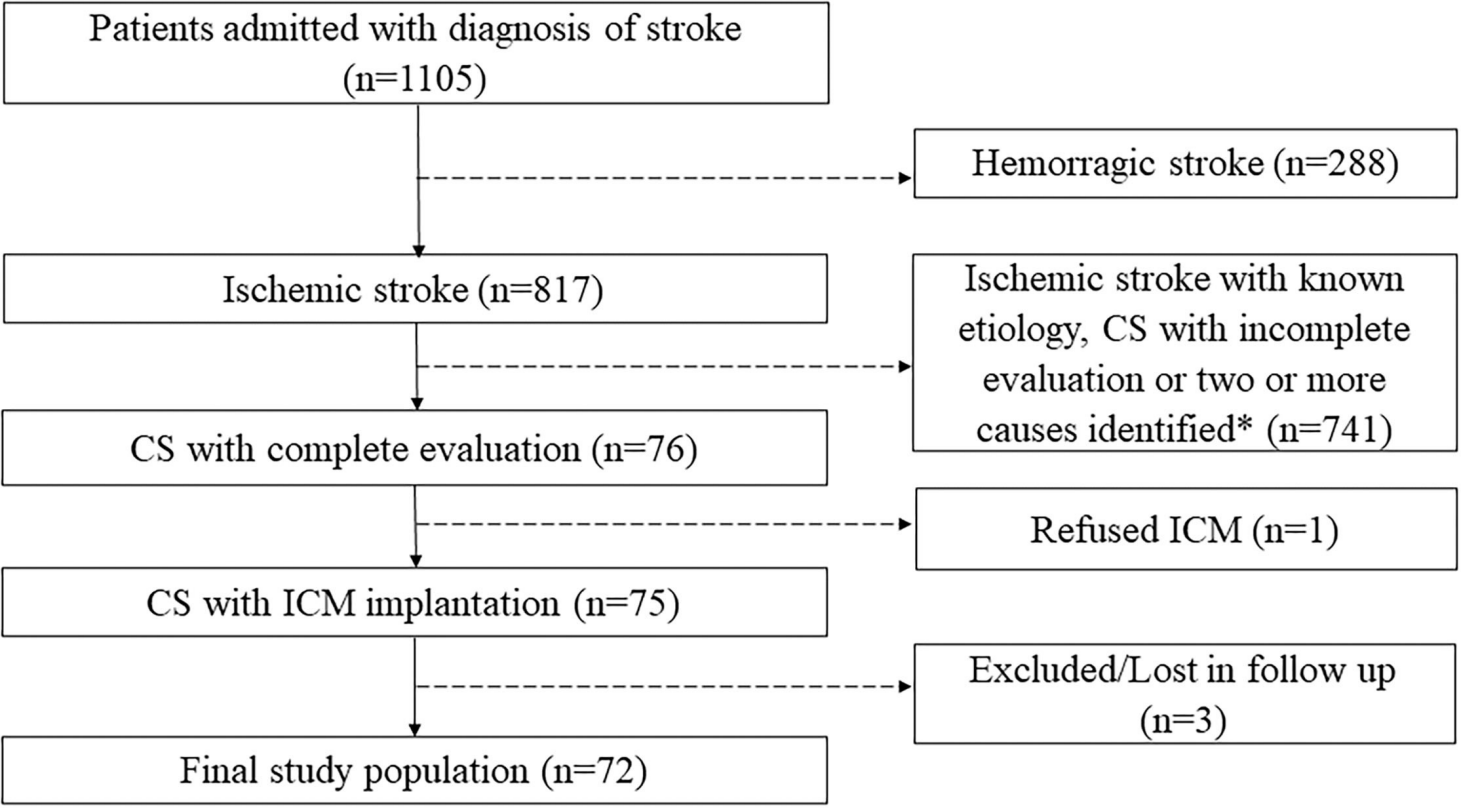
Flow diagram with inclusion and exclusion criteria. *According to Trial of Org 10172 in Acute Stroke Treatment's (TOAST's) classification. CS, cryptogenic stroke; ICM, insertable cardiac monitor.

Between March 2016 and September 2020, 1,105 patients were admitted for stroke to the Neurological Clinic of Santissima Annunziata Hospital of Chieti. CS was diagnosed in 76 of these, of which 75 underwent ICM implantation, and 72 completed follow up, constituting the final study population (Figure 1).

Mean age was 68 years, and most patients were males (59.4%) (Table 1). ICM were implanted, on average, at 3.6 ± 1 months (mean ± SD) after the index stroke. The mean follow-up period was 30.9 ± 1.9 months. Subclinical AF was detected in 23 patients (32%), on average, 6.5 ± 3.5 months after ICM implantation. A median of 10 subclinical AF episodes (IQR 3-234) lasting more than 2 min were recorded in AF group (Table 2).

**Table 1:** Demographic and clinical characteristics in the overall population and according to the occurrence of AF.

	**Overall**	**AF**	**No AF**	***p*-value**
	***n* = 72**	***n* = 23**	***n* = 49**	
Age—years, mean ± SD	67.7 ± 11.7	70.6 ± 12.6	66.4 ± 11.2	0.18
Female sex, *n* (%)	30 (41.6)	10 (43.5)	20 (40.8)	0.83
BMI, mean ± SD	27.5 ± 4.3	27.8 ± 4.9	27.4 ± 4.0	0.75
Hypertension, *n* (%)	49 (68)	17 (73.9)	32 (65.3)	0.46
Systolic blood pressure - mmHg, mean ± SD	122 ± 4	123 ± 8	121 ± 5	0.66
Diastolic blood pressure - mmHg, mean ± SD	75 ± 3	76 ± 10	75 ± 4	0.77
Diabetes mellitus, *n* (%)	17 (23.6)	6 (26)	11 (22.4)	0.73
Hypercholesterolemia, *n* (%)	53 (73.6)	12 (52.2)	41 (84)	**<0.01**
Coronary artery disease, *n* (%)	11 (15.3)	6 (23)	5 (10)	0.08
Valvular heart disease, *n* (%)	7 (9.7)	4 (17.4)	3 (6.1)	0.13
Cronic kidney disease, *n* (%)	6 (8)	3 (13)	3 (6)	0.29
Chronic obstructive pulmonary disease *n* (%)	9 (12.5)	3 (13)	6 (12.2)	0.92
Cancer/history of cancer, *n* (%)	11 (15.3)	4 (17.4)	7 (14.3)	0.73
Patent foramen ovale, *n* (%)	11 (15)	2 (8.7)	9 (18.4)	0.29
CHA2DS2-VASc, *n* (%)	5.1 (1.7)	5.8 (0.8)	4.7 (0.4)	**0.01**

*BMI, Body Mass Index; CHA2DS2-VASc, Congestive heart failure, Hypertension, Age ≥75 years, Diabetes mellitus, Stroke, Vascular disease, Age 65–74 years, Sex category (female). Statistically significant p-values are reported in bold*.

**Table 2:** Insertable cardiac monitor (ICM) data in the overall population and according to the occurrence of AF.

	**Overall**	**AF**	**No AF**
	***n* = 72**	***n* = 23**	***n* = 49**
Implantation of ICM after the index stroke (months)	3.6 (1)	2.5 (1.1)	4.1 (1.5)
Mean follow-up with ICM (months)	30.9 (1.9)	32.3 (3.6)	30.2 (2.3)
Diagnosis of AF after ICM implantation (months)		6.5 (3.5)	
AF burden (*n*)		10 (3–234)	

*Values are expressed as mean (SD), except AF burden expressed as median (IQR). AF, atrial fibrillation; ICM, Insertable Cardiac Monitor; AF Burden, number of AF episodes lasting more than 2 min*.

Patients with and without AF at ICM were homogeneous in all baseline characteristics, except for CHA_2_DS_2_-VASc score, which was significantly higher in AF group (5.8 ±.8 vs. 4.7 ±.4, *p* = 0.012) and for prevalence of hypercholesterolemia, which was significantly higher in no-AF group (84.0 vs. 52.2%, *p* = 0.005) (Table 1). In our cohort, patent foramen ovale (PFO) showed 15% of prevalence without significant differences between AF group and no AF group, although patients with PFO are numerically more frequent in the no-AF group. Furthermore, valvular heart disease (VHD) showed 9.7% of prevalence, without significant differences between the two groups. Table 3 shows the echocardiographic parameters significantly associated to the occurrence of AF at univariable logistic regression analysis.

**Table 3:** Echocardiographic parameters in the overall population and according to the occurrence of AF.

	**Overall**	**AF**	**No AF**	**Unadjusted OR**	***p*-value**	**Adjusted OR**	***p*-value**
	***n* = 72**	***n* = 23**	***n* = 49**				
LVEF (%)	63.0 (5.5)	60.0 (6.6)	64.4 (4.3)	0.85 (0.76–0.95)	**0.007**		
EDD (mm)	47.6 (5.8)	48.6 (6.0)	47.1 (5.7)		0.330		
SWT (mm)	10.2 (1.5)	10.4 (1.7)	10.2 (1.5)		0.690		
LVMI (g/m^2^)	90.6 (24.1)	93.4 (26.6)	89.4 (23.2)		0.560		
TAPSE (mm)	24.4 (3.3)	23.9 (3.1)	24.5 (3.4)		0.450		
LAES Area (cm^2^)	21.7 (4.5)	24.1 (5.1)	20.5 (3.8)	1.22 (1.06–1.39)	**0.006**		
LAVI (ml/m^2^)	34.5 (11.3)	40.4 (13.4)	31.7 (9.1)	0.01 (0.01–0.37)	**0.008**		
LATEF	0.5 (0.14)	0.4 (0.1)	0.5 (0.1)	0.01 (0.01–0.37)	**0.013**		
LAAEF	0.3 (0.1)	0.2 (0.1)	0.3 (0.1)		0.051		
LAPEF	0.3 (0.1)	0.2 (0.2)	0.3 (0.1)		0.080		
E velocity (cm/s)	76.3 (20.7)	83.7 (20.6)	72.9 (20.1)	1.03 (1.01–1.05)	**0.042**		
A velocity (cm/s)	86.9 (22.0)	89.1 (30.2)	85.9 (17.4)		0.650		
Mitral E velocity DT (ms)	236.2 (50.9)	253.3 (63.8)	228.4 (42.2)		0.100		
Mitral E/A ratio	0.9 (0.3)	1.0 (0.3)	0.9 (0.3)		0.100		
A duration (ms)	170.6 (3.7)	178.3 (31.3)	167.2 (37.3)		0.210		
PV S velocity (cm/s)	62.4 (12.6)	63.2 (12.2)	62.0 (12.9)		0.720		
PV D velocity (cm/s)	42.3 (11.9)	49.2 (15.7)	46.4 (9.7)		0.450		
PV AR velocity (cm/s)	38.1 (8.6)	35.7 (7.0)	39.3 (9.1)		0.100		
PV AR duration (ms)	167.7 (31.6)	182.9 (33.1)	160.6 (28.6)	1.02 (1.01–1.04)	**0.010**		
PV S/D	1.4 (0.4)	1.4 (0.5)	1.4 (0.3)		0.900		
Lateral TDI e'	9.4 (3.0)	9.0 (2.9)	9.6 (3.1)		0.410		
Septal TDI e'	6.6 (1.9)	6.4 (1.8)	6.6 (1.9)		0.570		
Mean TDI e'	8.0 (2.3)	7.7 (2.2)	8.2 (2.3)		0.420		
Lateral TDI E/e'	8.8 (3.5)	10.1 (4.1)	8.3 (3.1)		0.070		
Septal TDI E/e'	12.3 (4.5)	14.1 (5.3)	11.4 (3.9)	1.14 (1.01–1.27)	**0.045**		
Mean TDI E/e'	10.5 (3.8)	12.1 (4.4)	9.8 (3.2)	1.17 (1.02–1.35)	**0.039**		
PALS A4-C (%)	34.3 (14.5)	22.2 (10.7)	39.7 (12.7)	0.85 (0.78–0.92)	**<0.001**		
PALS A2-C (%)	29.0 (13.1)	23.4 (11)	31.8 (13.4)	0.93 (0.87–0.99)	**0.011**		
PACS A4-C^*^ (%)	15.0 (7.8)	8.0 (3.9)	18.2 (6.9)	0.70 (0.58–0.83)	**<0.001**	0.72 (0.48–0.90)	**0.005**
LA Conduit strain A4-C^*^ (%)	19.5 (2.3)	21.6 (2.8)	14.9 (3.5)	0.90 (0.82–0.98)	**0.005**		
LVLS A4-C^*^ (%)	−18.9 (3.9)	−16.6 (3.3)	−19.9 (3.7)	0.76 (0.63–0.91)	**0.001**	0.69 (0.46–0.95)	**0.041**

*Values are expressed as mean (SD)*.

*A4-C, apical four-chambers; A2-C, apical 2-chambers; AF, atrial fibrillation; LVEF, Left Ventricular Ejection Fraction; EDD, End Diastolic Left Ventricular Diameter; SWT, Septal Wall Thickness; LVMI, Left Ventricular Mass Index; LAES Area, Left Atrium End Systolic Area; LAVI, Left Atrial Volume Index; LATEF, Total Left Atrial Emptying fraction; LAAEF, Active Left Atrial Emptying Fraction; LAPEF, Passive Left Atrial Emptying Fraction; DT, Deceleration Time; PV, Pulmonary Vein; AR, Atrial Reversal; TDI, Tissue Doppler Imaging; PALS, Peak Atrial Longitudinal Strain; PACS, Peak Atrial Contractile Strain; LVLS, Left Ventricular Longitudinal Strain. Statistically significant p-values are reported in bold*.

* *For PACS, LA Conduit strain and LVLS, A4-C results are presented because of the better quality of the images*.

**Figure 2:**
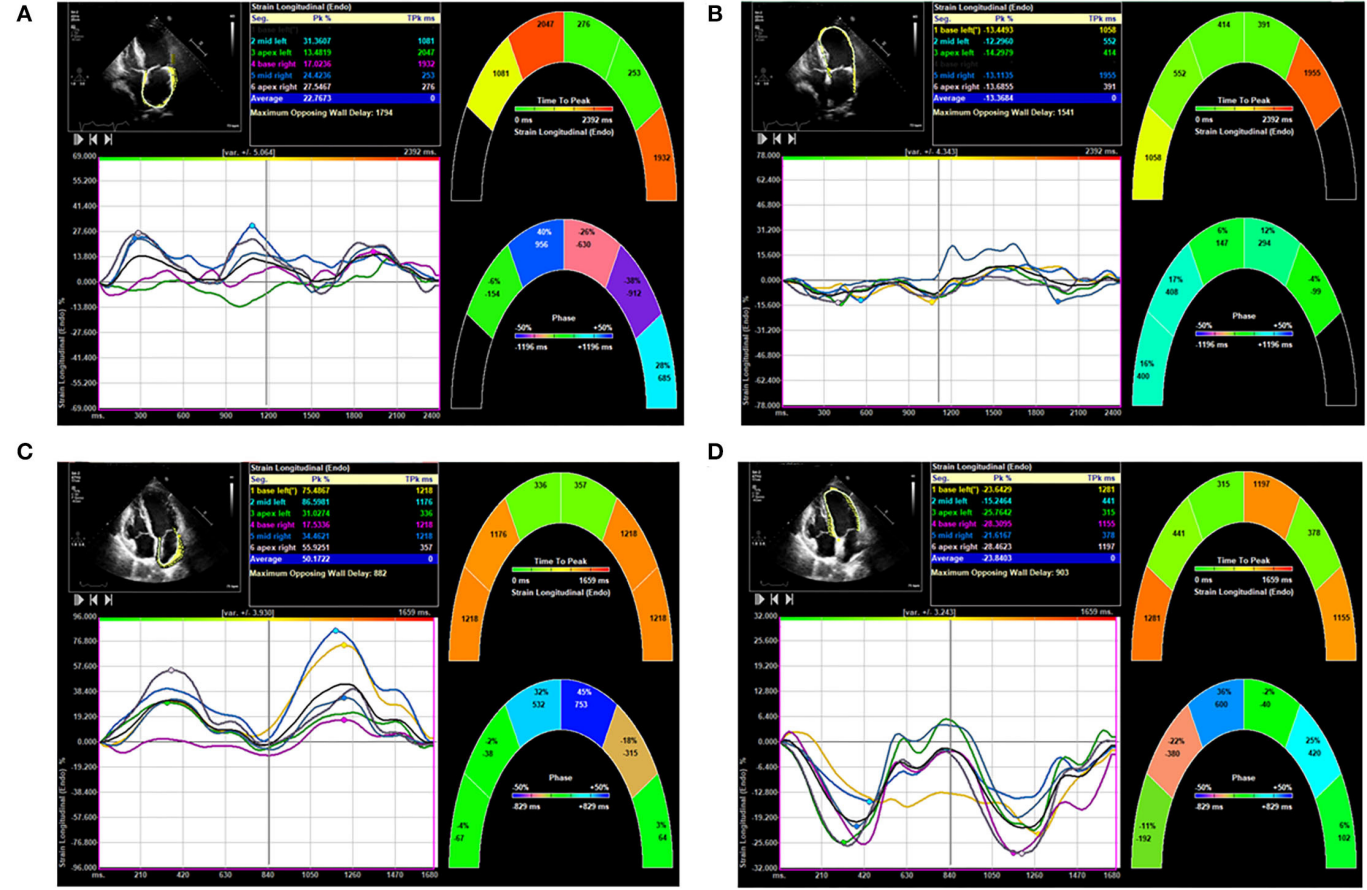
Comparison of left atrial (LA) and left ventricular (LV) strain in patients from atrial fibrillation (AF) group and no-AF group. **(A)** Shows LA strain of a patient of the AF group. **(B)** Shows LV strain of the same patient of the AF group. **(C)** Shows LA strain of a patient of the no-AF group. **(D)** Shows LV strain of the same patient of the no-AF group.

A comparison of LA and LV strain between an AF patient and no-AF patient is shown in Figure 2.

Multivariable regression analysis revealed that PACS and LV strain were the only parameters independently associated with AF (Table 3).

**Figure 3:**
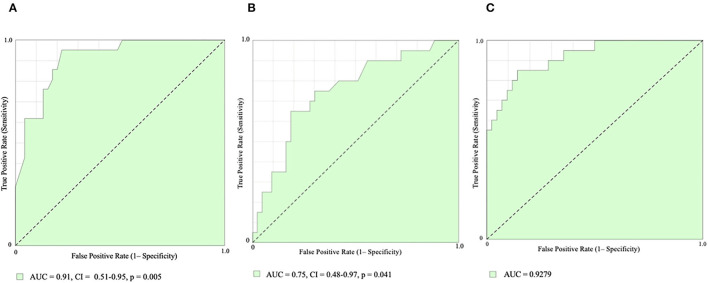
Receiver-operating characteristic (ROC) curve analysis. Peak Atrial Contractile Strain [PACS, **(A)**], Left Ventricular Longitudinal Strain [LVLS, **(B)**], and PACS combined with LVLS **(C)** for AF prediction. AUC, area under curve.

Receiver-operating characteristic (ROC) curve analysis showed that PACS had the best diagnostic performance (area under curve, AUC = 0.91, CI 0.51–0.95, *p* = 0.005; Figure 3A) for AF prediction, with a best cut-off value of 10.4% accounting for 86% of sensitivity and 76% of specificity. Also, LVLS showed good diagnostic performance (AUC 0.75, CI 0.48–0.97, *p* = 0.041; Figure 3B), with a best cut-off value of 16.9% accounting for 81% of sensitivity and 65% of specificity, and, in addition to PACS, led to significant improvements in AF prediction (AUC 0.92; Figure 3C).

**Figure 4:**
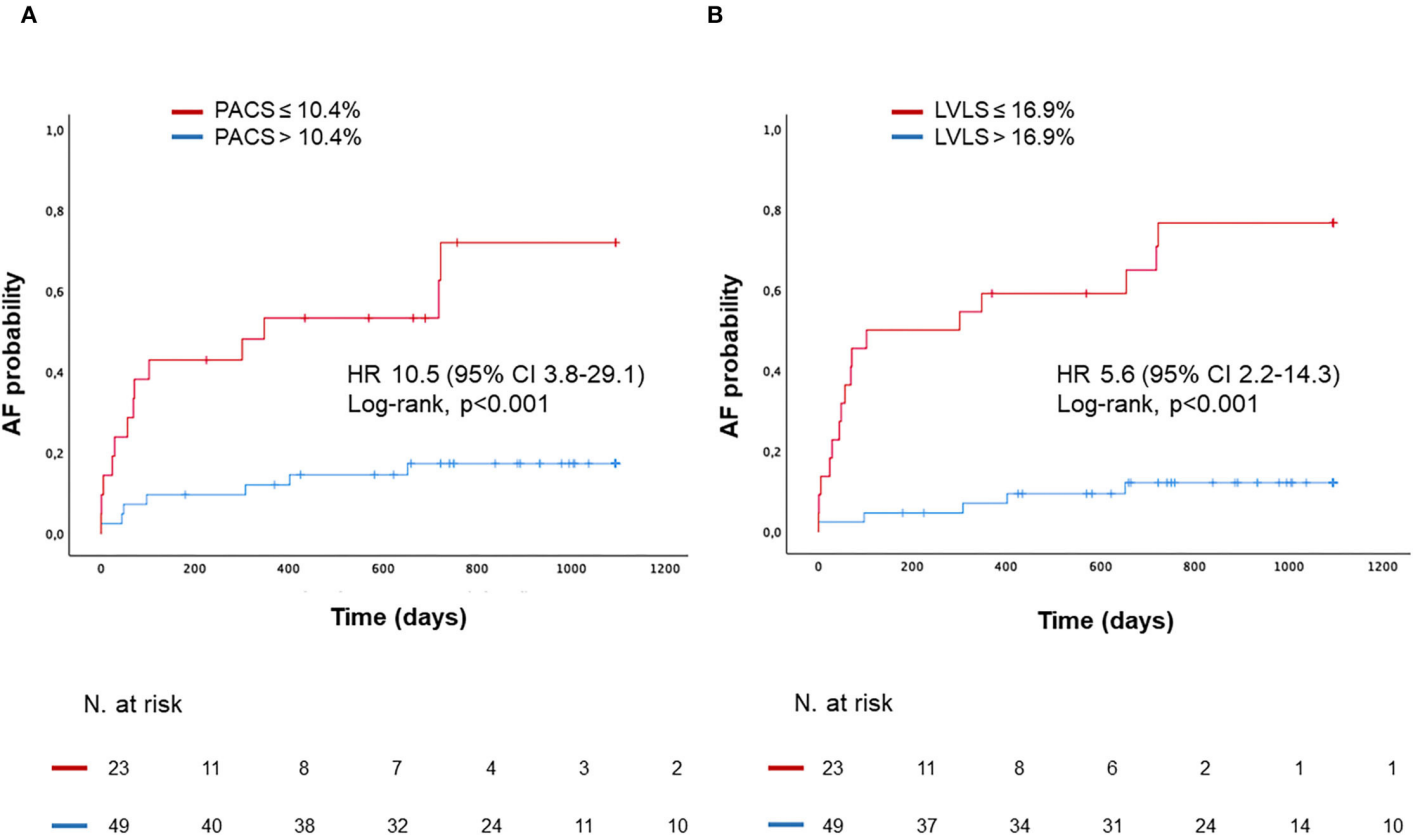
Kaplan–Meyer estimates of AF probability according to PACS **(A)**, or according to LVLS **(B)**. HR, Hazard Ratio.

Patients with impaired PACS and LVLS showed a much higher probability to develop AF during follow-up with a hazard ratio [HR 10.5 (95% CI 3.8–29.1) and HR 5.6 (95% CI 2.2–14.3) respectively, log-rank *P* < 0.001 for both; Figure 4].

PALS and PACS showed good inter and intra-observer correlation. Inter-observer correlation of PALS and PACS were expressed as Spearman's r of 0.70 (*p* = 0.003) and 0.69 (*p* = 0.004), respectively, and ICC was 0.74 and 0.61, respectively. Intra-observer correlation of PALS and PACS were expressed as Spearman's r of 0.7 (*p* = 0.004) and 0.7 (*p* = 0.004), respectively, and ICC was 0.76 and 0.78, respectively.

In our study, all patients presenting subclinical AF at follow-up underwent 12-lead ECG, of which only 3 of them manifested clinical AF in that occasion. However, all patients were prescribed oral anticoagulant therapy, considering their high thromboembolic risk and their preferences.

During a mean follow-up period of 30.9 ± 1.9 months, in the no-AF group, one acute myocardial infarction with persistent ST-segment elevation (STEMI) and one non-cardiovascular death were reported. Two recurrent strokes occurred in the AF group. In the first patient, the second stroke occurred before the implantation of ICM and consequently before the detection of AF. After the beginning of oral anticoagulation, no further strokes were detected. In the second patient, the additional stroke occurred under oral anticoagulation with dabigatran 110 mg bis in die (BID) after a de-escalation from dabigatran 150 mg BID driven by minor bleeding. After the second stroke, dabigatran 150 mg BID was restored, and no further strokes were detected. No major life-threatening or intracranial bleeding were reported in the entire study cohort.

## Discussion

There is an increasing interest in the potential role of subclinical AF in patients with CS. After several trials and confirmed by real-world data (10), last Guidelines from the European Society of Cardiology (ESC) on the management of AF (28) have set a Class IIa, level B indication for the use of ICMs after selected ischemic strokes in order to detect subclinical AF. ECG could improve risk stratification, identifying predictive parameters of AF and indicating patients worthy of receiving long-term ICM. In our study, almost one-third of patients with CS developed subclinical AF after on average about 6 months from ICM implantation. Occurrence of AF at long term follow-up was associated in our series by several echocardiographic parameters, including LA volume and LV systolic/diastolic function.

Among them, PACS and LVLS were the strongest and independent predictors of AF occurrence. PACS has been recently associated with AF in patients with CS without LA enlargement and emerged as a new useful echocardiographic parameter for the prediction of AF in this cohort of patients (22).

The value of this parameter, above LA sizing measurements, stands on the capability to detect even initial alteration of LA contractile function (22). Accordingly, in our analysis, LAVI and LA area, and also PALS, were good predictors of AF at univariable analysis, but their relevance was lost after adjustment for functional LA parameters, while PACS remained significantly associated with incident AF. Our findings are in line with previous studies. In fact, some series have shown an association between LA strain parameters and hidden AF in patients with CS (13, 21, 22). Particularly, PACS impairment can be considered a manifestation of atrial myopathy, being less dependent on LV mechanisms (22). This could explain its higher predictive value for the occurrence of AF in a setting of high-risk patients as those with cryptogenic stroke. Among other echocardiographic parameters, LVLS evaluates the deformation of the LV myocardium occurring during the cardiac cycle in the longitudinal plane, and it has been variously correlated to AF development and diastolic dysfunction (22, 29, 30). All conditions leading to diastolic dysfunction, including hypertension, diabetes, ischemic heart disease, and heart failure, could impair LA physiology (12, 17, 18, 31, 32) with a disease progression model that could encompass both atrial fibrosis and atrial mechanical dysfunction (11). Our results are also consistent with current evidence that proves that an impaired diastolic function in AF patients, as an expression of a close interaction between LA and LV function, results in an impaired LVLS (22, 29) and, probably, also reflects an impaired ventricular-arterial coupling (33, 34).

Among clinical parameters, age was not correlated to the occurrence of AF. Thus, suggesting that not the age *per se*, but age-related morpho-functional LA and LV dynamic changes are more important in this setting. CHA_2_DS_2_-VASc score was higher in patients who developed AF compared with those who did not, as previously observed in a population-based cohort of 22,179 middle-aged individuals (35). However, when adjusted for PACS and LVLS, it lost its predictive value.

We have also found a higher prevalence of hypercholesterolemia in patients without AF at long term follow-up. One may speculate about a non-cardioembolic cause of CS in such patients, with a prevailing atherosclerotic etiology of the stroke. Similarly, the higher number, although not significant, of patients with PFO in the no-AF group could lead to consider this as related to the stroke.

In our study, all patients presenting subclinical AF at follow-up were prescribed oral anticoagulant therapy, considering their high thromboembolic risk and their preferences. Although the routine use of oral anticoagulation (OAC) in subclinical AF remains a matter of debate, in the last ESC Guidelines (28), the use of OAC may be considered in selected patients according to AF burden and according to individual risk of stroke, expressed as CHA_2_DS_2_-VASc score. In our study, the AF group had a mean CHA_2_DS_2_-VASc score of 5.8. For this reason, the estimated high individual risk of stroke led clinicians to start OAC administration also in presence of subclinical AF.

Further large series studies are needed to address clinical implications of the predictive value of echocardiographic parameters, including PACS and LVLS, for the occurrence of AF. Since the research of hidden AF is crucial in patients with CS and can lead to an adequate therapeutic regimen, availing of predictive parameters of subclinical AF might indicate patients worthy of receiving long-term ICM. Furthermore, LA and LV strain is useful to find out the atrial myopathy and dysfunction related to AF and often preceding the occurrence of the arrhythmia. Therefore, these echocardiographic parameters can be exploited to identify higher risk patients in which the research of AF is strongly recommended, including patients with CS, but also in other high-risk clinical settings.

## Strength and Limitations

To our knowledge, this is the largest study with the totality of CS population monitored using ICM. In fact, most studies were conducted on patients monitored with conventional methods, while only one enrolled 56 patients with CS monitored with ICM (13).

There are some limitations in this study. First, this is a single-center study with a small sample size. Therefore, a correlation of clinical and echocardiographic variables with hard clinical events was not possible. Moreover, since the ICM here used requires at least 2 min of AF to be detected, episodes <2 min could have been missed, while definition of AF now accounts for episodes lasting more than 30 s with absence of P waves and variable R-R interval. Furthermore, 15 patients were unable to attend a clinical visit, and follow-up data were collected by a phone questionnaire. Finally, for some strain data, only A4-C view results are presented because of the better quality of the images. However, although in most studies, the 2D-global longitudinal strain was calculated as the average of the A4-C, A2-C, and A3-C views, the A4-C view is considered adequate to obtain strain measures when the echocardiographic acoustic window is limited (26).

## Conclusions

In about one-third of patients with CS, ICM revealed subclinical AF episodes. In these patients, LA and LV strain analysis adds predictive value for the occurrence of AF over clinical and other morpho-functional echocardiographic parameters. Impaired booster pump strain and left ventricular longitudinal strain are strong and independent predictors of AF.

## Data Availability Statement

The original contributions presented in the study are included in the article/supplementary materials, further inquiries can be directed to the corresponding author/s.

## Ethics Statement

The studies involving human participants were reviewed and approved by Institutional Ethics Committee of G. d'Annunzio University/Santissima Annunziata Hospital of Chieti. The patients/participants provided their written informed consent to participate in this study.

## Author Contributions

GB and GR contributed to the conception and design of the work. GB, FR, CD'A, and FP contributed to the acquisition and analysis of data for the work. GB, FR, SG, GR, and SP contributed to the interpretation of data. GB and FR drafted the manuscript. MD, MF, SP, SG, and GR critically revised the manuscript. All authors gave final approval and agreed to be accountable for all aspects of work ensuring integrity and accuracy.

## Conflict of Interest

FR: speaker/consultant fee from Boehringer Ingelheim, Daiichi Sankyo. SP: speaker/consultant fee from Astra Zeneca, Bayer. GR: speaker/consultant fee from Astra Zeneca, Bayer, BMS-Pfizer, Boehringer Ingelheim, Daiichi Sankyo. The remaining authors declare that the research was conducted in the absence of any commercial or financial relationships that could be construed as a potential conflict of interest.

## Publisher's Note

All claims expressed in this article are solely those of the authors and do not necessarily represent those of their affiliated organizations, or those of the publisher, the editors and the reviewers. Any product that may be evaluated in this article, or claim that may be made by its manufacturer, is not guaranteed or endorsed by the publisher.
